# Analysis of factors influencing pancreatic fistula after minimally invasive pancreaticoduodenectomy and establishment of a new prediction model for clinically relevant pancreatic fistula

**DOI:** 10.1007/s00464-024-10770-6

**Published:** 2024-03-18

**Authors:** Yuwen Zhu, Di Wu, Hao Yang, Zekun Lu, Zhiliang Wang, Guangchen Zu, Zheng Li, Xiaowu Xu, Yue Zhang, Xuemin Chen, Weibo Chen

**Affiliations:** 1 Department of Hepatopancreatobiliary Surgery, The First People’s Hospital of Changzhou, The Third Affiliated Hospital of Soochow University, Changzhou, China; 2 Department of Pancreatic Surgery, Fudan University Shanghai Cancer Center, Shanghai, China

**Keywords:** Minimally invasive pancreaticoduodenectomy, Postoperative pancreatic fistula, Risk factors, Prediction model

## Abstract

**Background:**

Postoperative pancreatic fistula (POPF) is the most prevalent complications following minimally invasive pancreaticoduodenectomy (MIPD). Only one model related to MIPD exists, and previous POPF scoring prediction methods are based on open pancreaticoduodenectomy patients. Our objectives are to determine the variables that may increase the probability of pancreatic fistula following MIPD and to develop and validate a POPF predictive risk model.

**Methods:**

Data from 432 patients who underwent MIPD between July 2015 and May 2022 were retrospectively collected. A nomogram prediction model was created using multivariate logistic regression analysis to evaluate independent factors for POPF in patients undergoing MIPD in the modeling cohort. The area under the curve (AUC) of the receiver operating characteristic curve (ROC) and the calibration curve were used to verify the nomogram prediction model internally and externally within the modeling cohort and the verification cohort.

**Results:**

Multivariate logistic regression analysis showed that body mass index (BMI), albumin, triglycerides, pancreatic duct diameter, pathological diagnosis and intraoperative bleeding were independent variables for POPF. On the basis of this information, a model for the prediction of risks associated with POPF was developed. In accordance with the ROC analysis, the modeling cohort's AUC was 0.819 (95% CI 0.747–0.891), the internal validation cohort's AUC was 0.830 (95% CI 0.747–0.912), and the external validation cohort's AUC was 0.793 (95% CI 0.671–0.915). Based on the calibration curve, the estimated values of POPF have a high degree of concordance with the actual values that were measured.

**Conclusions:**

This model for predicting the probability of pancreatic fistula following MIPD has strong predictive capacity and can provide a trustworthy predictive method for the early screening of high-risk patients with pancreatic fistula after MIPD and timely clinical intervention.

Pancreaticoduodenectomy (PD) is the main surgical method for treating both benign and malignant tumors of the pancreatic head, duodenal papilla, ampulla of Vater and lower common bile duct [1, 2]. The surgery for these tumors includes resection and reconstruction. Because of the multiple organs and the complex digestive tract reconstruction, it is considered as one of the most complex operations in general surgery [3, 4]. Laparoscopic and robotic PD are both components of the MIPD surgical technique. MIPD has been gradually introduced in numerous pancreatic centers worldwide over the past two decades. When compared to regular PD, MIPD has the advantages of causing less trauma during surgery, allowing for a quicker recovery after the procedure, and requiring a shorter postoperative stay duration in the hospital [5, 6]. However, the international questioning of MIPD has not stopped, mainly due to the long learning curve, the incidence of postoperative complications being similar to that of open pancreaticoduodenectomy (OPD), and the higher mortality [7].

Following PD, one of the most prevalent complications that carries a high-risk is POPF, which can occur in 5–40% of PD patients [8]. POPF can lead to serious complications, including bleeding and infection, which can increase the duration of hospitalization and cost, delay postoperative adjuvant treatment, and increase the risk of death [9]. With the constant advancement of surgical techniques, the mortality rate of PD surgery has decreased significantly [10]. However, over the past 30 years, the incidence of POPF has remained basically stable even in major pancreatic disease centers worldwide [11, 12].

Although there are a variety of pancreatic fistula risk scoring systems, there are still many deficiencies in the application of MIPD. First, most of the pancreatic fistula scoring systems were established based on the study of POPF in patients after OPD, and data of MIPD patients were not included [9, 13]. The difference between this study and previous studies is that the aims of this study are to determine the risk variables associated with POPF, to improve the assessment of the probability of POPF by creating a nomogram that accounts for the aforementioned risk variables and to establish and verify a new predictive risk model for pancreatic fistula after MIPD.

## Methods

### Study design and patient selection

Data from 432 patients who underwent MIPD between July 2015 and May 2022 in the Third Affiliated Hospital of Soochow University and Fudan University Shanghai Cancer Center were retrospectively acquired and divided into a modeling cohort, an internal validation group and an external verification cohort. The following are the criteria for inclusion in the list: 1. All patients underwent MIPD surgery; 2. No distant metastases were detected by preoperative imaging; and 3. No additional combination resections for malignant tumors were performed. The exclusion criteria were as follows: 1. Incomplete clinical data or loss of follow-up of the patients; 2. Neoadjuvant therapy was administered to patients prior to surgery; 3. Patients with severe underlying diseases who were unable to tolerate surgery; and 4. Patients were solely given palliative care. The Ethics Committee gave their blessing to proceed with this study. The clinical records of all of the patients who participated in this study submitted their signed informed approval, and the data were incorporated into the study. The work has been reported in line with the STROCSS criteria [14].

### Data collection

In this study, a series of perioperative data were collected, including general physiological conditions, preoperative biochemical indicators, intraoperative conditions and histopathological parameters. Age, sex, BMI, smoking history, history of diabetes and the presence or absence of obstructive jaundice, cholangitis, and biliary drainage prior to surgery were among the basic clinical and background information. The preoperative examination included preoperative white blood cells (WBCs), platelets, hemoglobin, neutrophil-to-lymphocyte count ratio (NLR), alanine aminotransferase (ALT), aspartate aminotransferase (AST), alkaline phosphatase (ALP), Na^+^, K^+^, total bilirubin (TB), direct bilirubin (DB), total protein, albumin, triglyceride, cholesterol and international normalized ratio (INR). Histopathology and intraoperative data comprised the American Society of Anesthesiologists (ASA) score, pathologic diagnosis, pancreatic texture, pancreatic duct diameter, operation time, and volume of intraoperative bleeding.

### Surgical approach

The operations were carried out in accordance with standard protocols and by two surgeons with extensive experience in pancreatic surgery. The feasibility, safety and scope of the operations were determined by imaging examinations before the operation.

For LPD, the surgical procedure was described in our previous article [15, 16]. The patients were placed in the supine position with legs apart. After general anesthesia and tracheal intubation, laparoscopic exploration was conducted to rule out peritoneal and visceral surface metastases. The gastrocolic ligament was divided below the gastroepiploic artery arch with ultrasonic sheers, and the right gastroepiploic artery was transected. The fourth portion of the duodenum and proximal jejunum was mobilized, and the jejunum was transected 15 cm distal to the Treitz ligament with a linear stapler. The stump of the jejunum was dragged into the supramesocolic compartment. The stomach was divided proximal to the antrum with a linear stapler. The superior mesenteric vein (SMV) was identified, and a tunnel was dissected anterior to the SMV and portal vein (PV). The GDA and right gastric artery were clipped and divided. The pancreatic neck parenchyma was divided by an ultrasonic dissector, and the main pancreatic duct was identified and divided with scissors. The uncinate process was exposed at the left posterior aspect of the SMA. The uncinate process was dissected with an ultrasonic dissector along the right border of the SMA, and the tributary vessels were identified, clipped and dissected. The common hepatic artery and the hepatic artery were isolated and hung by tape. Both cholecystectomy and common hepatic bile duct transection were performed, and the corresponding lymph nodes were harvested. The specimens were entirely removed. A duct-to-mucosa pancreaticojejunostomy was performed. An end-to-side choledochojejunostomy was performed using a continuous suture and was strengthened with two stitches at the two corners. The gastrojejunostomy was performed antecolic or retrocolic with a linear stapler, and the common opening was closed to complete the reconstruction. Three abdominal drainage tubes were placed behind the anastomotic site of pancreaticojejunostomy and choledochojejunostomy and at the right side of the inferior vena cava. RPD was conducted with a da Vinci Xi surgical system. The dissection and reconstruction procedures were similar to those of LPD.

### Classification and definition of postoperative pancreatic fistula

A pancreatic fistula is diagnosed when the amylase content of the drainage fluid is greater than three times the upper limit of the normal value of serum amylase at more than three days after surgery, as defined by the International Study Group of Pancreatic Surgery (ISGPS) in 2016 [17, 18]. POPF classification: Biochemical leak (BL) is a past that of a grade A fistula. B-grade pancreatic fistula affects the postoperative process, and the expected postoperative management needs to be changed. Grade C is defined as organ dysfunction affecting one or more organs, reoperation, or postoperative mortality from pancreatic fistula infection. The ISGPS-developed classification rationale and treatment guidelines were used to grade POPF in this study. Grade B and grade C pancreatic fistulas were identified as having clinical significance in this study [17].

### Statistical analyses

For the entire cohort, all data were examined for normality using the Kolmogorov‒Smirnov test. The mean and standard deviation of continuous variables with a normal distribution were calculated, and Student’s t test was used to examine the data. The Mann‒Whitney U test was used to assess non-normally distributed data, which are expressed as the median (interquartile range). The Pearson chi-square test was used to assess the dichotomous variables, and percentages were used to represent the results. In the subsequent multivariate analysis using logistic regression, only the variables with *P* < 0.05 in the univariate analysis were considered significant. The risk prediction model was created using R software using the factors that had *P* < 0.05 in the multivariate analysis. The specificity and sensitivity of the model were assessed in this study using the AUC of the ROC and its 95% CI, and the calibration curve was utilized to illustrate the correlation between the observed and predicted incidence. All statistical analyses were carried out by utilizing IBM SPSS Statistics (version 26.0, IBM Corp.) and R Studio (version 4.3.1, Vienna, Austria).

## Results

### The baseline data

A total of 432 patients who had undergone MIPD were included in this study. Of these patients, 200 were assigned to the modeling cohort, 110 patients were included in the internal validation cohort, and 122 patients were included in the external validation cohort. The cohort for modeling included 112 men, with an average age of 66.1 ± 9.8 years. The internal validation cohort included 68 men, who were on average 66.8 ± 11.0 years old. The clinical data from the two cohorts, including general and biochemical data, did not significantly differ between the cohorts (*P* > 0.05, Table 1) and were comparable. A total of 16.0% (32/200) of the modeling cohort and 18.2% (20/110) of the validation cohort experienced CR-POPF.

**Table 1:** Demographic and clinical characteristics in the modeling cohort and validation cohort

Variable	Modeling cohort (*n* = 200)	Validation cohort (*n* = 110)	*P* value
Age (years)	66.1 ± 9.8	66.8 ± 11.0	0.660
Sex, male, *n* (%)	112 (56.0)	68 (61.8)	0.321
BMI (kg/m^2^)	23.0 ± 2.7	23.2 ± 3.1	0.190
Smoke, *n* (%)	23 (11.5)	10 (9.1)	0.511
Diabetes mellitus, *n* (%)	37 (18.5)	28(25.5)	0.150
WBC (10^9^/L)	5.9 (4.6–7.1)	5.5 (4.5–7.2)	0.746
Platelet (10^9^/L)	227.6 ± 77.1	233.0 ± 73.1	0.655
Hemoglobin (g/L)	124.6 ± 17.4	122.6 ± 17.4	0.661
NLR	2.8 (2.0–4.2)	3.2 (2.5–4.4)	0.091
ALT (μ/L)	62.5 (20.0–141.5)	76.5 (33.6–215.7)	0.105
AST (μ/L)	39.0 (20.6–101.0)	45.0 (33.7–142.8)	0.147
ALP (μ/L)	199.0 (90.3–407.1)	300.0 (164.3–490.1)	0.214
Na + (mmol/L)	140.3 ± 3.3	138.1 ± 3.1	0.853
K + (mmol/L)	4.1 ± 0.5	3.8 ± 0.5	0.449
TB (μmol/L)	23.4 (10.3–110.8)	31.1 (15.7–141.5)	0.269
DB (μmol/L)	14.5 (4.1–91.9)	23.6 (7.1–121.4)	0.113
Total protein (g/L)	64.2 ± 7.8	65.0 ± 7.3	0.329
Albumin (g/L)	37.0 ± 5.5	36.7 ± 5.5	0.575
Triglyceride (mmol/L)	1.6 (1.1–2.5)	1.9 (1.2–2.7)	0.167
Cholesterol (mmol/L)	5.7 ± 2.0	6.0 ± 2.5	0.108
INR	1.0 ± 0.1	1.0 ± 0.1	0.835
Pancreatic duct (mm)	3.7 ± 1.6	4.0 ± 1.0	0.109
Pathology, *n* (%)			0.598
PDAC or pancreatitis	65 (32.5)	39 (35.5)	
Other than PDAC or pancreatitis	135 (67.5)	71 (64.5)	
Pancreatic texture, n (%)			0.289
Hard	13 (6.5)	4 (3.6)	
Soft	187 (93.5)	106 (96.4)	
Intraoperative bleeding, (mL)	200.0 (100.0–337.5)	160.0 (100.0–400.0)	0.203
ASA class, *n* (%)			0.363
I	3 (1.5)	1 (0.9)	
II	141 (70.5)	69 (62.7)	
III	55 (27.5)	40 (36.4)	
IV	1 (0.5)	0 (0.0)	
Operative time (min)	357.0 ± 87.2	332.5 ± 67.7	0.083
Preoperative obstructive jaundice, *n* (%)	108 (54.0)	65 (59.1)	0.388
Preoperative cholangitis, n (%)	24 (12.0)	8 (7.3)	0.191
Preoperative biliary drainage, *n* (%)	25 (12.5)	11 (10.0)	0.511
Postoperative pancreatic fistula, n (%)	32 (16.0)	20 (18.2)	0.623

*BMI* body mass index, *WBC* white blood cell, *NLR* neutrophil-to-lymphocyte ratio, *ALT* alanine aminotransferase, *AST* aspartate aminotransferase, *ALP* alkaline phosphatase, *TB* total bilirubin, *DB* direct bilirubin, *INR* international normalized ratio, *PDAC* pancreatic ductal adenocarcinoma, *ASA* American Society of Anesthesiologists score

### Risk factors related to pancreatic fistula in the modeling set

According to the univariate analysis, as indicated in Table 2, BMI ≥ 24 kg/m^2^, WBC > 10 × 10^9^/L, albumin < 35 g/L, triglyceride ≥ 1.7 mmol/L, intraoperative bleeding ≥ 400 mL, pancreatic duct ≤ 3 mm and pathological diagnosis except pancreatic ductal adenocarcinoma (PDAC) or pancreatitis were significantly associated with POPF (*P* < 0.05). Other factors and the appearance of pancreatic fistula following MIPD were not found to be significantly related.

**Table 2:** Univariate and multivariate analysis results of risk factors related to POPF after MIPD

Variable	Univariate analysis		Multivariate analysis	
	OR (95.0% CI)	*P*	OR (95.0% CI)	*P*
Age (> 65)	1.033 (0.991–1.076)	0.127		
Sex (male)	0.726 (0.334–1.580)	0.420		
BMI (≥ 24 kg/m^2^)	2.860 (1.216–6.729)	0.016	2.479 (1.081–7.762)	0.034
Smoke (yes)	2.143 (0.477–9.629)	0.320		
Diabetes mellitus (yes)	2.453 (0.705–8.534)	0.158		
WBC (> 10 × 10^9^/L)	2.495 (1.090–5.710)	0.030	3.166 (1.143–8.776)	0.068
Platelet (< 100 × 10^9^/L)	0.586 (0.215–1.597)	0.420		
Hemoglobin (< 110 g/L)	1.601 (0.678–3.781)	0.283		
ALT (≥ 56μ/L)	1.123 (0.521–2.422)	0.767		
AST (> 40μ/L)	1.226 (0.573–2.624)	0.600		
TB (≥ 20.5 μmol/L)	1.308 (0.613–2.790)	0.488		
Albumin (< 35 g/L)	2.576 (1.181–5.618)	0.017	3.114 (1.088–7.283)	0.031
Triglyceride (≥ 1.7 mmol/L)	2.293 (1.034–5.085)	0.041	2.695 (1.553–6.034)	0.019
Cholesterol (> 5.2 mmol/L)	1.556 (0.714–3.390)	0.538		
Preoperative obstructive jaundice (yes)	1.211 (0.568–2.580)	0.621		
Preoperative cholangitis (yes)	1.057 (0.336–3.329)	0.924		
Preoperative biliary drainage (yes)	0.817 (0.228–1.877)	0.673		
Pancreatic duct (≤ 3 mm)	3.322 (1.136–7.627)	0.003	4.145 (1.283–7.571)	0.008
Pathology (other than PDAC or pancreatitis)	3.000 (1.098–8.096)	0.032	3.738 (1.167–8.285)	0.028
Pancreas texture (Soft)	1.071 (0.226–5.081)	0.931		
Intraoperative bleeding (≥ 400 mL)	3.667 (1.076–6.445)	0.036	4.695 (1.126–9.742)	0.025
ASA class (< 3)	0.596 (0.269–1.319)	0.202		
Operative time (≥ 350 min)	1.211 (0.565–2.593)	0.622		

*BMI* body mass index, *WBC* white blood cell, *ALT* alanine aminotransferase, *AST* aspartate aminotransferase, *TB* total bilirubin, *PDAC* pancreatic ductal adenocarcinoma, *ASA* American Society of Anesthesiologists score

Multivariate logistic regression was applied in this work to further evaluate the data. At the multivariate level, this study identified six risk predictors of pancreatic fistula after MIPD. The findings indicated that BMI ≥ 24 kg/m^2^ (OR 2.479, 95% CI 1.081–7.762, *P* = 0.034), albumin < 35 g/L (OR 3.114, 95% CI 1.088–7.283, *P* = 0.031), triglycerides ≥ 1.7 mmol/L (OR 2.695, 95% CI 1.553–6.034, *P* = 0.019), intraoperative bleeding ≥ 400 mL (OR 4.695, 95% CI 1.126–9.742, *P* = 0.025), pancreatic duct ≤ 3 mm (OR 4.145, 95% CI 1.283–7.571, *P* = 0.008) and pathological diagnosis except PDAC or pancreatitis (OR 3.738, 95% CI 1.167–8.285, *P* = 0.028) were predictors of POPF (Table 2). Pancreatic fistula and white blood cell count were not significantly correlated with one another (*P* > 0.05).

### Establishment and verification of the nomogram prediction model

**Fig. 1:**
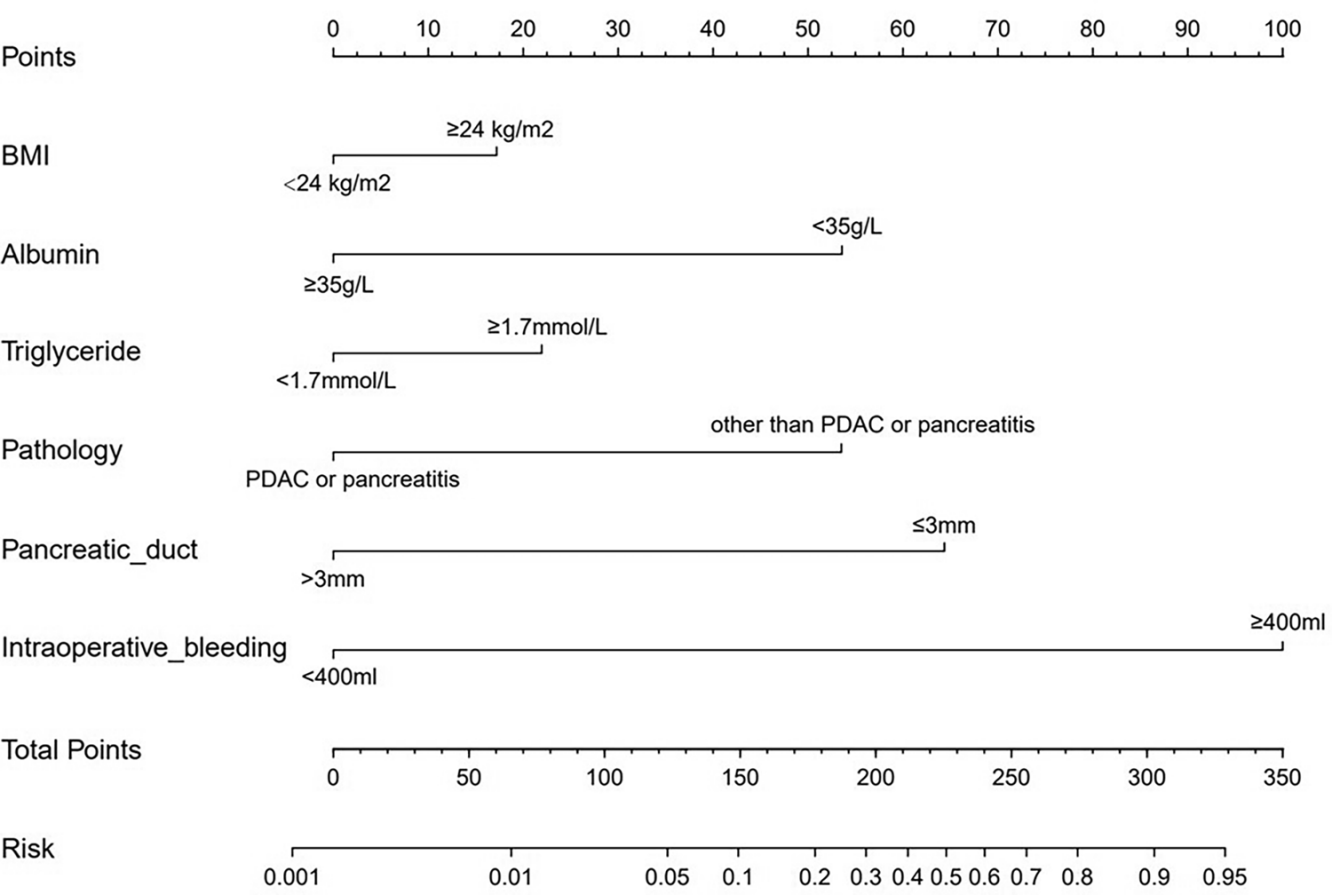
Nomogram calculator in prediction for postoperative pancreatic fistula after minimally invasive pancreaticoduodenectomy

The statistically significant risk factors identified by the multivariate analysis, including high BMI, low albumin, high triglyceride, small pancreatic duct diameter, pathological diagnosis except PDAC or pancreatitis, and intraoperative bleeding, were included in the nomogram to help predict the risk of POPF in patients with MIPD. The nomogram showed that a narrow pancreatic duct and substantial intraoperative bleeding had the greatest impact on the score. For each variable, values in the range of 0 to 100 in the chart are proportionally assigned according to the variable’s regression coefficient of its associated with POPF. Through the scale above the model, the individual scores corresponding to the six independent risk factors can be obtained, and one's overall score is calculated by summing all of their individual scores. Patients who have MIPD have a risk of POPF that corresponds to the prediction probability related to their overall score (Fig. 1).

**Fig. 2:**
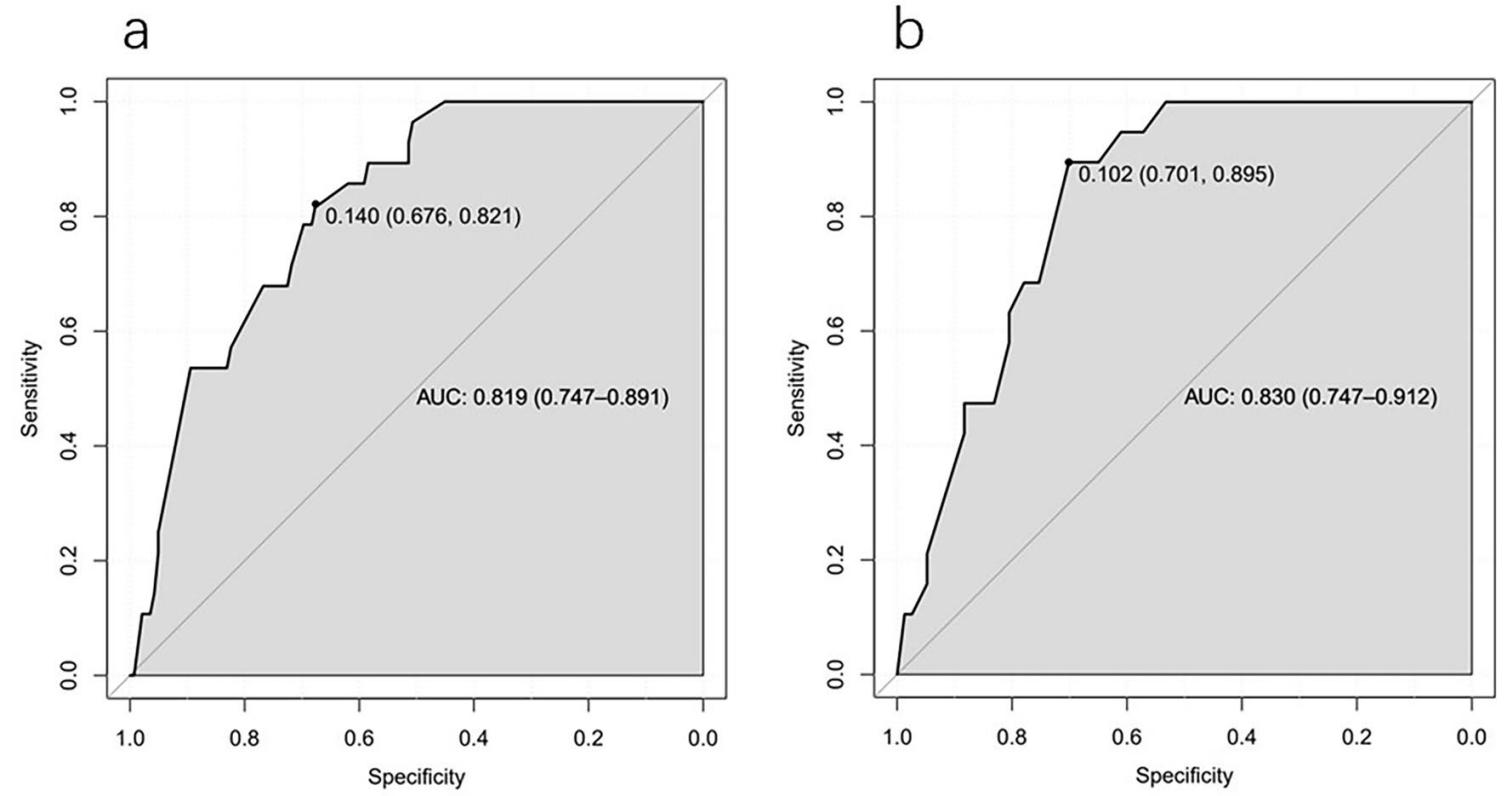
Receiver operating characteristic (ROC) curve of predictive model in modeling cohort (**a**) and validation cohort (**b**)

**Fig. 3:**
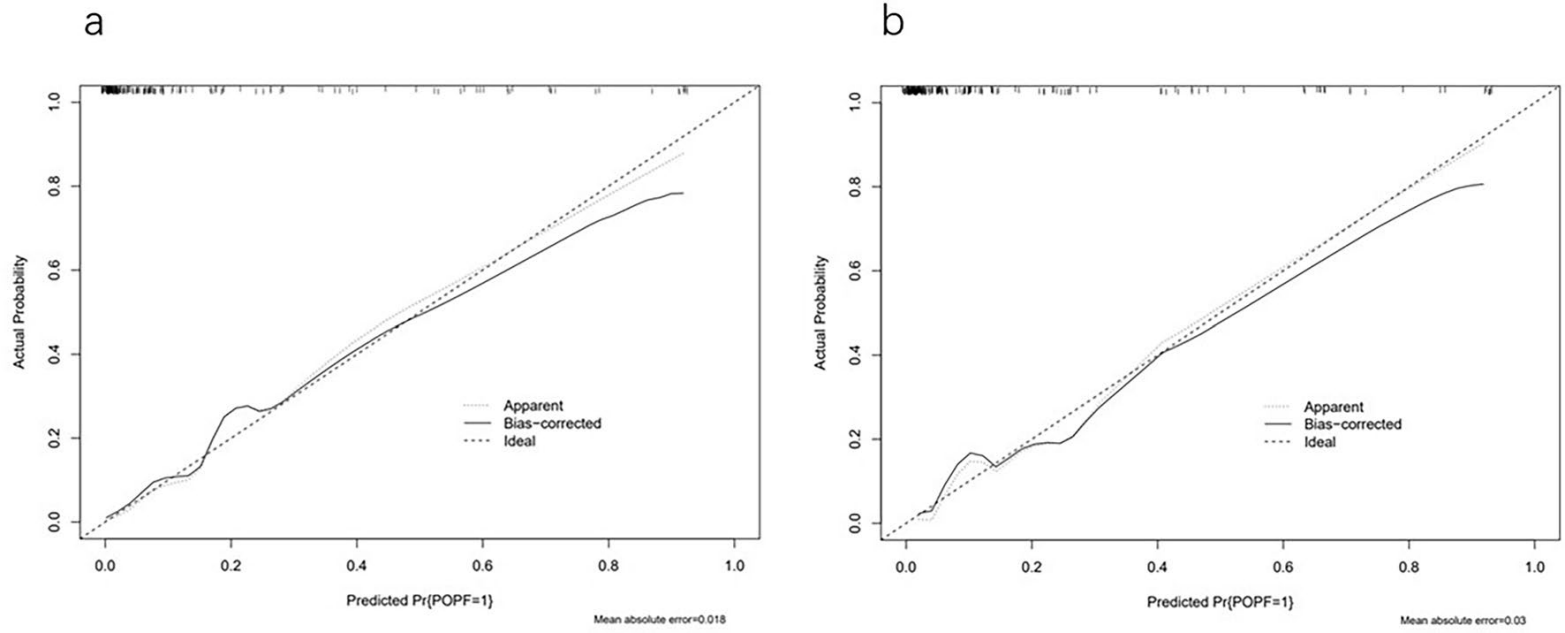
Calibration curves for the modeling cohort (**a**) and validation cohort (**b**)

**Fig. 4:**
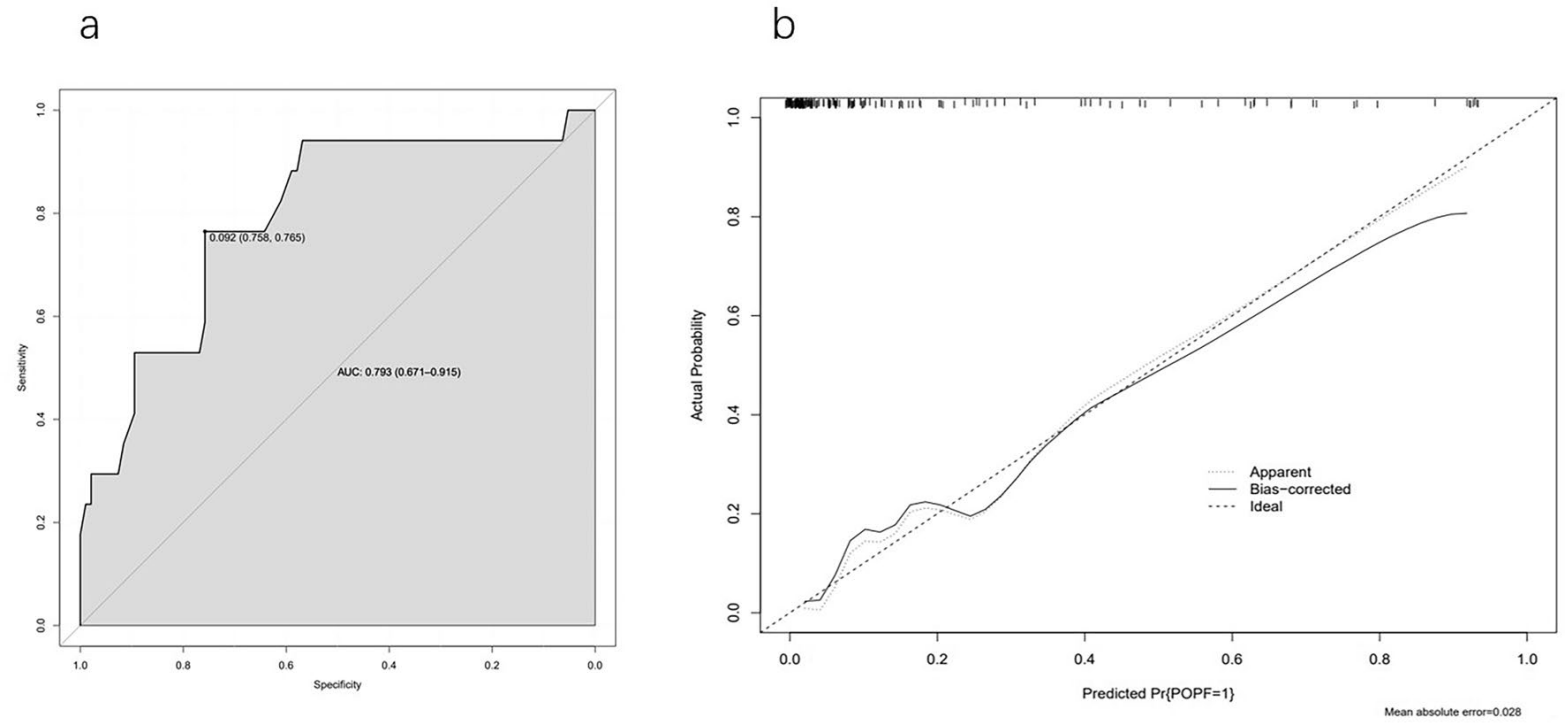
Receiver operating characteristic (ROC) curve (**a**) and calibration curve (**b**) of the external validation cohort

Then, ROC analysis was performed on the prediction model. The modeling cohort's AUC was 0.819 (95% CI 0.747–0.891), and the internal validation cohort's AUC was 0.830 (95% CI 0.747–0.912). The AUC values were all greater than 0.8, indicating that the prediction model had a high-level of discrimination (Fig. 2). In addition, we conducted verifications of the established prediction model. The calibration curve results demonstrated that the mean absolute errors (MAEs) of the two cohorts were 0.018 and 0.03, respectively (the smaller the MAE value was, the higher the calibration degree), demonstrating a greater calibration degree for the prediction model (Fig. 3). On this basis, we also conducted an external validation of the model. The AUC of the external validation cohort was 0.793 (95% CI 0.671–0.915), and the calibration curve showed that MAEs was 0.028 (Fig. 4). The results demonstrated that the actual findings were consistent with the projected risk of POPF.

## Discussion

We found that BMI, albumin, triglyceride, pancreatic duct diameter, pathological diagnosis, and intraoperative bleeding were independent variables affecting POPF. Based on these data, a model was developed to predict the risk of POPF. The model has a strong predictive ability to predict the probability of pancreatic fistula after MIPD, which can provide a reliable prediction method for early screening and timely clinical intervention of high-risk patients with pancreatic fistula after MIPD.

The most effective therapy for improving the prognosis of ampullary cancer, lower common bile duct cancer, pancreatic head cancer, and other periampullary malignant tumors at this time is surgery in combination with chemotherapy [2]. After pancreaticoduodenectomy, serious consequences are possible, with pancreatic fistula being one of the most frequent. Examining the causes of pancreatic fistula and possible treatments is very beneficial from a clinical standpoint. However, studies reveal that between 10 and 40% of patients who undergo pancreaticoduodenectomy will develop a pancreatic fistula [19–21], and although medical standards, surgical procedures, and perioperative treatment approaches are always being improved, the development of a pancreatic fistula seriously affects the short-term prognosis of patients, prolongs hospitalization time, increases hospitalization costs, and leads to postoperative systemic inflammatory response, failure of multiple organs, and even mortality, causing serious health threats. Although many studies have found indicators of risk for complications following PD, few studies have constructed predictive models for predicting complications after MIPD. Most of the pancreatic fistula scoring systems were established based on the study of POPF in patients after OPD, and data of MIPD patients were not included [9, 13]. The updated alternative fistula risk score (ua-FRS) was the only POPF prediction model using the MIPD cohort [22]. Some medical centers are now gradually using MIPD as the main surgical method, and so is our center. Results showed that the incidence of POPF in patients with MIPD remained at a normal level, similar to the international incidence of POPF after PD, and did not increase significantly [7, 22, 23]. Therefore, our study only involved patients undergoing MIPD and did not include data from OPD patients. The objective is to establish and verify a new risk prediction model for pancreatic fistula-based entirely on MIPD patients to comprehensively evaluate the indicators of risk for POPF following MIPD and provide a more reliable prediction tool for clinicians to screen high-risk patients and thus intervene early.

In a study of the related risk factors for POPF in patients with pancreaticoduodenectomy, age, sex, preoperative jaundice, BMI, operation time, intraoperative bleeding, pathological diagnosis, pancreatic duct and pancreatic texture were reported as risk factors [24–27]. However, according to the findings of our research, sex, age, and pancreatic texture were not associated with the prediction of POPF. Additionally, multivariate logistic regression analysis demonstrated that intraoperative bleeding, pathological diagnosis, pancreatic duct, albumin, triglyceride, and BMI were all independent predictors of pancreatic fistula following pancreaticoduodenectomy.

First, in terms of BMI, patients with a high BMI have been reported in earlier research to have a higher risk of problems following PD [26, 28]. Taking Edoardo Rosso et al.'s study as an example [29], which was a retrospective analysis of 111 cases, it was discovered that having a high BMI was a significant contributor to the chance of developing POPF. Our research summary considers the following reasons. 1. The pancreas becomes more difficult to access during surgery when there is a greater amount of fat both within the abdominal cavity and surrounding the pancreas; 2. Because the pancreas is fat-rich and delicate, there is a considerable risk of pancreatic injury during surgery. 3. During pancreaticojejunostomy, fatty pancreatic tissue and the pancreatic duct are more likely to be injured during suturing and knotting, thereby increasing the risk of pancreatic fistula.

You et al. found that low preoperative albumin constituted a separate risk factor for POPF [23]. Huang et al. stated that compared with patients with preoperative albumin ≥ 30 g/L, patients with albumin < 30 g/L had a higher risk of POPF [30]. According to the results of a multivariate logistic regression analysis, the difference was statistically significant, indicating that preoperative albumin ≥ 30 g/L was a factor that decreased the possibility of developing POPF following pancreaticoduodenectomy. Our results are consistent with their research. Studies have found that decreased albumin levels increase the risk of POPF, but it does not mean that POPF does not occur in patients with normal albumin levels.

A small pancreatic duct diameter is recognized as a potential cause of pancreatic fistula [31]. Patients with a small pancreatic duct diameter had a higher probability of developing POPF following PD than patients with a large pancreatic duct diameter, according to a univariate analysis that revealed the difference to be statistically significant. In addition, the difference was still statistically significant after applying multivariate logistic regression analysis, suggesting that a small pancreatic duct diameter was a separate risk factor for POPF following PD. The OR (4.145, 95% CI 1.283–7.571) demonstrated that patients with a pancreatic duct diameter had a 4.145-times higher probability of developing a pancreatic fistula following PD than those with a large pancreatic duct diameter.

Finally, this study examined the relationship between triglycerides, pathological diagnosis and intraoperative blood loss and pancreatic fistula after MIPD. Previous studies have shown that high triglycerides and intraoperative blood loss predict a greater likelihood of pancreatic fistula following PD [32–34], while individuals with a pathological diagnosis of pancreatic cancer or pancreatitis have a noticeably decreased incidence of pancreatic fistula following PD [13]. Our findings are consistent with this, suggesting that triglycerides, pathological diagnosis and intraoperative blood loss are the factors of POPF after MIPD.

Certain restrictions apply to this study. First, our study data from a single center is lack of diversity, and the number of patients and events is relatively small. Second, all operations were performed by two surgeons with rich experience in pancreatic surgery, and their personal surgical habits may have a certain bias on the results of the study. Finally, although the prediction model has certain innovative significance, there are also limitations, such as an insufficient number of research objects and observation variables, which can have a certain impact on the research results, so the results need to be further verified by subsequent clinical trials.

In summary, the nomogram prediction model established in this study has high accuracy. The clinicopathological characteristics, as well as the physical and chemical indications that can be easily collected in MIPD patients, were used to develop the nomogram model of POPF. This model can be useful in the early prediction and risk assessment of POPF in patients receiving MIPD. In our opinion, early prediction of POPF does not mean that we can prevent the occurrence of POPF. This prediction model may provide more favorable clinical guidance for surgeons, and we can take measures to minimize the consequences of POPF. The most common surgical complications leading to patient death are uncontrolled repeated bleeding and intra-abdominal infection caused by a grade C pancreatic fistula; thus, the leading task is to prevent grade C pancreatic fistulas [9]. When performing MIPD in a patient with a high-risk of POPF, we may conduct the procedure more precisely and with more caution and take actions to reinforce the pancreatojejunostomy or modify the pancreatojejunostomy technique. We can use the greater omentum or the round ligament of the liver to separate the stump of the GDA from the anastomotic site of the pancreaticojejunostomy to prevent possible erosion of the GDA stump and late hemorrhage. We could also place two drainage tubes at the superior and inferior borders of the pancreaticojejunostomy site and prolong the removal time of the drainage tube. However, the benefits of the prediction model in guiding surgeons when performing MIPD need further verification by randomized clinical trials.
